# A Retrospective Cohort Study Evaluating the Use of the Modified Early Warning Score to Improve Outcome Prediction in Neurosurgical Patients

**DOI:** 10.7759/cureus.28558

**Published:** 2022-08-29

**Authors:** Michael Karsy, Joshua C Hunsaker, Forrest Hamrick, Matthew N Sanford, Amanda Breviu, William T Couldwell, Devin Horton

**Affiliations:** 1 Department of Neurosurgery, University of Utah, Salt Lake City, USA; 2 School of Medicine, University of Utah, Salt Lake City, USA; 3 Department of Strategic Initiatives, University of Utah, Salt Lake City, USA; 4 Department of Internal Medicine, University of Utah, Salt Lake City, USA

**Keywords:** outcomes, outcome prediction, decompensation, mews, neurosurgery, modified early warning score

## Abstract

Introduction

The modified early warning score (mEWS) has been used to identify decompensating patients in critical care settings, potentially leading to better outcomes and safer, more cost-effective patient care. We examined whether the admission or maximum mEWS of neurosurgical patients was associated with outcomes and total patient costs across neurosurgical procedures.

Methods

This retrospective cohort study included all patients hospitalized at a quaternary care hospital for neurosurgery procedures during 2019. mEWS were automatically generated during a patient’s hospitalization from data available in the electronic medical record. Primary and secondary outcome measures were the first mEWS at admission, maximum mEWS during hospitalization, length of stay (LOS), discharge disposition, mortality, cost of hospitalization, and patient biomarkers (i.e., white blood cell count, erythrocyte sedimentation rate, C-reactive protein, and procalcitonin).

Results

In 1,408 patients evaluated, a mean first mEWS of 0.5 ± 0.9 (median: 0) and maximum mEWS of 2.6 ± 1.4 (median: 2) were observed. The maximum mEWS was achieved on average one day (median = 0 days) after admission and correlated with other biomarkers (p < 0.0001). Scores correlated with continuous outcomes (i.e., LOS and cost) distinctly based on disease types. Multivariate analysis showed that the maximum mEWS was associated with longer stay (OR = 1.8; 95% CI = 1.6-1.96, p = 0.0001), worse disposition (OR = 0.82, 95% CI = 0.71-0.95, p = 0.0001), higher mortality (OR = 1.7; 95% CI = 1.3-2.1, p = 0.0001), and greater cost (OR = 1.2, 95% CI = 1.1-1.3, p = 0.001). Machine learning algorithms suggested that logistic regression, naïve Bayes, and neural networks were most predictive of outcomes.

Conclusion

mEWS was associated with outcomes in neurosurgical patients and may be clinically useful. The composite score could be integrated with other clinical factors and was associated with LOS, discharge disposition, mortality, and patient cost. mEWS also could be used early during a patient's admission to stratify risk. Increase in mEWS scores correlated with the outcome to a different degree in distinct patient/disease types. These results show the potential of the mEWS to predict outcomes in neurosurgical patients and suggest that it could be incorporated into clinical decision-making and/or monitoring of neurosurgical patients during admission. However, further studies and refinement of mEWS are needed to better integrate it into patient care.

## Introduction

Early warning scores are vital sign-based scoring systems that have been used in hospital settings to help identify patients whose condition is deteriorating and automatically alert staff [1-6]. Early identification of decompensating patients may allow for more prompt interventions, potentially leading to better outcomes and safer, more cost-effective patient care [1-6]. Described by Stenhouse et al. in 1999 as a modification of the early warning system by Morgan et al., the modified early warning score (mEWS) was developed for the real-time detection of sepsis decompensation in hospitalized adults from operative or emergency room settings [2,7-11]. A score of >4 was recommended to flag patients for additional review. Escobar et al. describe the conceptual framework for early detection of patient decline using biometric data rather than standard clinical judgment to alter overall patient care trajectories with the earlier intervention [2]. Multiple studies since the development of mEWS and other scoring systems have shown efficacy in triaging septic and nonseptic patients in an emergency department, inpatient, and critical care settings [2,7-11].

Although an increasing mEWS has been predictive of general surgery postoperative complications [12], to our knowledge, no study has evaluated postoperative complications in a neurosurgical population. One study of mEWS included neurosurgical patients as part of a larger cohort of sepsis patients and showed that automatically generated alerts could reduce poor patient outcomes and costs by prompting earlier care [13]. However, there has been no specific subanalysis of mEWS in neurosurgical patients, which are distinct from other patient populations. The purpose of this study was to evaluate whether the use of the mEWS in monitoring and treatment of neurosurgical patients can be predictive of patient outcomes. We assessed the predictive value of the score for discharge disposition, length of stay (LOS), mortality, and total patient costs across various neurosurgical procedures.

## Materials and methods

Patients

Institutional Review Board approval with a waiver of informed consent was acquired for retrospective analysis of a prospectively generated database. All hospitalized neurosurgical patients treated at a single tertiary-care center in 2019 were included in the analysis. As previously described [13] software was built into the electronic health record (EHR) that would automatically calculate a vital sign-based mEWS any time vital signs were entered by the nursing staff (Table 1). Development and implementation of the EHR software involved a lead-in time involving staff training. If a complete set of vital signs was not entered, the EHR would redirect the staff back to complete the collection of vital signs. The mEWS thresholds were set based on internal mortality data.

**Table 1:** Factors included in the modified early warning score (mEWS)

Score	3	2	1	0	1	2	3
Temperature (°C)		≤35.0	35.1–35.5	35.6–38.0	38.1–39.0	39.1–40.9	≥40.0
Respiratory rate	≤8		9–11	12–20	21–25	26–29	≥30
Pulse	≤30	31–39		40–100	101–110	111–130	≥131
Systolic blood pressure	≤80	81–90	91–100	101–180	181–200	201–220	≥221

All visit data that were documented in the EHR were later transferred to the academic center Enterprise Data Warehouse (EDW), an Oracle-based database (Redwood City, CA) that allowed the compilation of vital signs, patient demographic, and outcome data. Patients included in this study were neurosurgical elective and nonelective inpatients, patients from January 2019 to December 2019, and patients with available clinical and mEWS data in the EDW. Patient procedures were noted based on Current Procedural Terminology (CPT) coding. Admission site, whether the critical care or general floor, was noted. All of the mEWS data for each patient’s hospital visit were evaluated. The first mEWS on the day of admission were recorded. The maximum mEWS, defined as the highest recorded mEWS during the patient’s hospitalization, was noted. Patients were categorized into the following neurosurgery diagnosis subcategories: spine, vascular, trauma, tumor, peripheral nerve, functional, and other, based on CPT codes and admitting surgeons. Patient demographics, clinical biomarkers, and the Charlson Comorbidity Index (CCI) were also collected from the EDW. Our search parameters in the EDW resulted in 1,408 patient evaluations, which were reviewed for accuracy. Based on available patient coding at the time of analysis, patients were aggregated into clinical categories.

Patient costs were obtained using the value-driven outcome (VDO) tool, which has been described previously [14,15]. Costs measured by the VDO tool are direct care costs attributable to patient care activities at the level of individual patient encounters; thus, they are not patient charges, cost-to-charge ratios, or reimbursements, which are considered to be less accurate [15]. Although true costs cannot be reported because of the sensitivity of such data, costs were summed for the group, and the proportion of an individual patient’s cost is reported as a percentage to allow for comparison between patient groups.

Patient outcomes included discharge disposition, LOS, mortality, and cost. Routine disposition was defined as a home or home-health disposition. Longer LOS and higher cost were defined as greater than the 50th percentile (median) for the entire group.

Statistics

Data means with standard deviations are reported for continuous data. Numbers and percentages of totals are reported for discrete data. Continuous data were analyzed by one-way analysis of variance with Tukey post-hoc correction whereas discrete data were analyzed by chi-squared testing. Pearson correlation was performed for the mEWS and biomarkers, namely, C-reactive protein (CRP), erythrocyte sedimentation rate (ESR), white blood cell (WBC) count, and procalcitonin level. Statistical analysis was performed by SPSS (V23.0, IBM Corp., Armonk, NY). Predictive logistic regression nomograms and various machine learning algorithms were used to analyze patient data to predict outcomes using Orange software (University of Ljubljana, Ljubljana, Slovenia) [16]. The predictive normogram used a logistic regression classifier to generate score weights. Default settings were used for machine learning analysis: neural networks used a multi-layer perceptron supervised algorithm via scikit-learn version 0.24.1 (sklearn.neural_network.MLPClassifier) with default settings, and support vector machines used the LIBSVM algorithm (www.csie.ntu.edu.tw/~cjlin/libsvm/). All other methods (e.g., naïve Bayes, tree, and random forest) used Orange software defaults (orangedatamining.com).

## Results

Demographics

**Figure 1:**
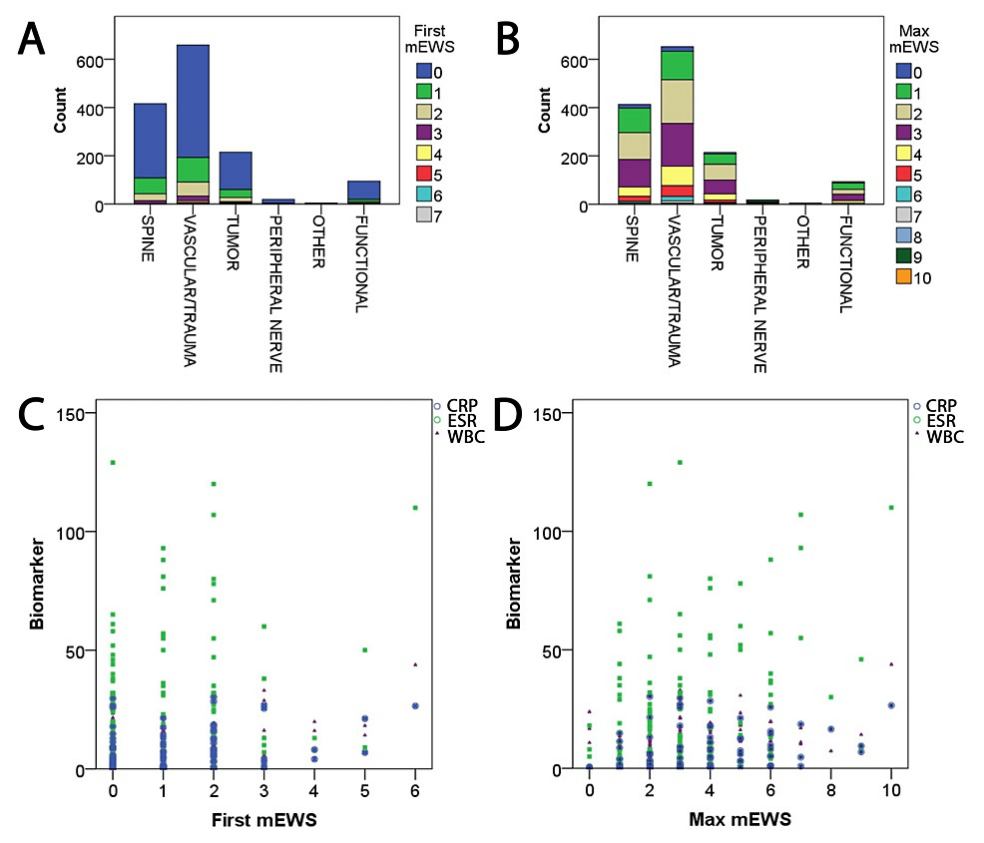
Evaluation of first and maximum mEWS values The (A) first and (B) maximum mEWS are demonstrated across different treatment categories. (C) The first mEWS correlated with CRP (r = 0.4, p = 0.0001), ESR (r = 0.29, p = 0.0001), and WBC count (r = 0.34, p = 0.0001). (D) The maximum mEWS correlated with CRP (r = 0.34, p = 0.0001), ESR (r = 0.33, p = 0.0001), and WBC count (r = 0.23, p = 0.0001). Procalcitonin was not included in the correlation with the mEWS because of insufficient data. mEWS, modified early warning score; CRP, C-reactive protein; ESR, erythrocyte sedimentation rate; WBC, white blood cell.

A total of 1,408 neurosurgical patients were evaluated, including spine (416), vascular/trauma (659), tumor (215), peripheral nerve (19), functional (95), and other categories (four) (Table 2). Patient baseline information is presented in Table 3. The mean CCI was 2.6 ± 2.7 (median: 2.0). The first mEWS score (mean: 0.5 ± 0.9, median: 0, range: 0-7.0) and the maximum mEWS score (mean: 2.5 ± 1.4, median: 2.0, range: 0-10.0) were determined; these were also calculated for the subspecialty groups separately (Table 2, Figures 1A, 1B). The mean amount of time required to reach the maximum mEWS score was 1 ± 3 days (median: 0 days, range: 0-30 days), and the mean LOS was 6.04 ± 6.96 days (median: 3.97 days, range: 0-72.5 days). Other common biomarkers of infection and potential sepsis were recorded, including first WBC count (mean: 10.0 ± 4.5, median: 9.25), first ESR (mean: 22.7 ± 26.2, median: 13.0), first CRP (mean: 4.9 ± 7.4, median: 1.0), and first procalcitonin level (mean: 0.9 ± 3.5, median: 1.0). The first and maximum mEWS were shown to correlate with the biomarkers (p < 0.0001; Figures 1C, 1D).

**Table 2:** Patient outcomes associated with the modified early warning score (mEWS) LOS, length of stay; AMA, against medical advice; SNF, skilled nursing facility.

Variable	All (n = 1408)	Spine (n = 416)	Vascular/trauma (n = 659)	Tumor (n = 215)	Peripheral nerve (n = 19)	Functional (n = 95)	Other (n = 4)	P-value
First mEWS (SD)	0.5 (0.9)	0.4 (0.8)	0.5 (1.0)	0.5 (1.0)	0.4 (1.0)	0.3 (0.8)	0.5 (1.0)	0.3
Max mEWS (SD)	2.5 (1.4)	2.4 (1.4)	2.7 (1.5)	2.6 (1.4)	2.3 (1.4)	2.2 (1.3)	2.8 (2.1)	0.007
Time to max mEWS, days (SD)	1 (3)	1.0 (2.2)	1.7 (3.3)	1.4 (2.8)	0.8 (2.1)	0.5 (1.2)	2.0 (6.2)	0.0001
Outcomes								
Mean LOS (SD)	6.04 (6.96)	5.18 (5.45)	7.11 (8.45)	5.68 (5.11)	4.36 (3.14)	5.5 (5.77)	3.5 (3.71)	0.0001
Total (mean % of cost) (%)	0.071 (0.0718)	0.0638 (0.0543)	0.0803 (0.0883)	0.056 (0.0453)	0.0442 (0.026)	0.0553 (0.0236)	0.0785 (0.0556)	0.0001
Disposition (%)								
Rehabilitation	208 (14.8)	57 (13.7)	93 (14.2)	46 (21.5)	5 (26.3)	7 (7.4)	0 (0)	0.001
AMA	10 (0.7)	4 (1)	3 (0.5)	3 (1.4)	0 (0)	0 (0)	0 (0)	
Law enforcement	7 (0.5)	5 (1.2)	2 (0.3)	0 (0)	0 (0)	0 (0)	0 (0)	
Death/hospice	32 (2.3)	1 (0.2)	23 (3.5)	8 (3.7)	0 (0)	0 (0)	0 (0)	
Another facility	24 (1.7)	5 (1.2)	15 (2.3)	4 (1.9)	0 (0)	0 (0)	0 (0)	
Home/home health	1009 (71.9)	296 (71.2)	471 (71.8)	139 (65.0)	14 (73.7)	85 (89.5)	4 (100)	
SNF	114 (8.1)	48 (11.5)	49 (7.5)	14 (6.5)	0 (0)	3 (3.2)	0 (0)	

**Table 3:** Patient baseline data * includes leukemia and lymphoma. CCI, Charlson Comorbidity Index; AIDS/HIV, acquired immune deficiency syndrome/human immunodeficiency virus; CHS, congestive heart failure; MI, myocardial infarction; WBC, white blood cell; ESR, erythrocyte sedimentation rate; CRP, C-reactive protein; AMA, against medical advice; SNF, skilled nursing facility.

Variable	All (n = 1408)	Spine (n = 416)	Vascular/trauma (n = 659)	Tumor (n = 215)	Peripheral nerve (n = 19)	Functional (n = 95)	Other (n = 4)	P-value
Mean age, years (SD)	54.5 (17.6)	59.1 (14.5)	54.2 (18)	51.4 (18)	54.4 (22.5)	43.5 (18.2)	40.5 (23.8)	0.0001
Mean CCI (SD)	2.6 (2.7)	2.4 (2.6)	2.6 (2.7)	3.6 (3.2)	2.9 (3.3)	1.2 (1.5)	2.3 (2.1)	0.0001
No. of males (%)	765 (54)	223 (53.6)	354 (53.7)	118 (54.9)	13 (68.4)	53 (55.8)	4 (100)	0.4
No. with comorbidities (%)								
AIDS/HIV	3 (0.2)	1 (0.2)	2 (0.3)	0 (0)	0 (0)	0 (0)	0 (0)	0.96
Non-metastatic malignancy*	205 (14.7)	38 (9.2)	64 (9.8)	96 (44.9)	1 (5.6)	5 (5.4)	1 (25)	0.0001
Cerebrovascular disease	612 (43.9)	93 (22.5)	409 (62.6)	74 (34.6)	2 (11.1)	33 (35.5)	1 (25)	0.0001
Chronic pulmonary disease	305 (21.9)	111 (26.9)	136 (20.8)	38 (17.8)	3 (16.7)	17 (18.3)	0 (0)	0.06
CHF	114 (8.2)	32 (7.7)	70 (10.7)	11 (5.1)	0 (0)	1 (1.1)	0 (0)	0.005
Dementia	53 (3.8)	12 (2.9)	27 (4.1)	8 (3.7)	1 (5.6)	5 (5.4)	0 (0)	0.8
Diabetes w/o chronic complication	139 (10)	44 (10.7)	76 (11.6)	14 (6.5)	0 (0)	5 (5.4)	0 (0)	0.09
Diabetes w/ chronic complication	131 (9.4)	51 (12.3)	53 (8.1)	23 (10.7)	0 (0)	4 (4.3)	0 (0)	0.05
Hemi-/paraplegia	169 (12.1)	45 (10.9)	83 (12.7)	23 (10.7)	9 (50.0)	6 (6.5)	3 (75.0)	0.0001
Metastatic solid-tumor malignancy	96 (6.9)	30 (7.3)	24 (3.7)	39 (18.2)	2 (11.1)	1 (1.1)	0 (0)	0.0001
Mild liver disease	123 (8.8)	31 (7.5)	60 (9.2)	25 (11.7)	3 (16.7)	4 (4.3)	0 (0)	0.2
Moderate or severe liver disease	2 (0.1)	0 (0)	2 (0.3)	0 (0)	0 (0)	0 (0)	0 (0)	0.8
MI	123 (8.8)	34 (8.2)	64 (9.8)	21 (9.8)	2 (11.1)	2 (2.2)	0 (0)	0.2
Peptic ulcer disease	41 (2.9)	16 (3.9)	21 (3.2)	3 (1.4)	1 (5.6)	0 (0)	0 (0)	0.3
Peripheral vascular disease	178 (12.8)	51 (12.3)	99 (15.2)	22 (10.3)	2 (11.1)	4 (4.3)	0 (0)	0.05
Renal disease	119 (8.5)	32 (7.7)	69 (10.6)	13 (6.1)	3 (16.7)	2 (2.2)	0 (0)	0.03
Rheumatic disease	71 (5.1)	32 (7.7)	27 (4.1)	6 (2.8)	1 (5.6)	5 (5.4)	0 (0)	0.08
First WBC (SD)	10.0 (4.5)	9.7 (3.9)	10.2 (4.7)	11.2 (5.2)	8.1 (3.3)	7.3 (2.7)	14.0 (8.2)	0.1
First ESR (SD)	22.7 (26.2)	29.0 (31.1)	19.8 (23.9)	26.9 (25.9)	5.5 (7.8)	11.2 (18.5)	4.5 (3.5)	0.2
First CRP (SD)	4.9 (7.4)	6.8 (8.3)	4.1 (6.7)	4.8 (8.3)	2.4 (3.0)	1.5 (3.3)	11.9 (1.3)	0.2
First procalcitonin (SD)	0.9 (3.5)	2.1 (6.5)	0.5 (2.0)	1.6 (5.6)	–	–	–	0.5

Multivariate analysis

**Figure 2:**
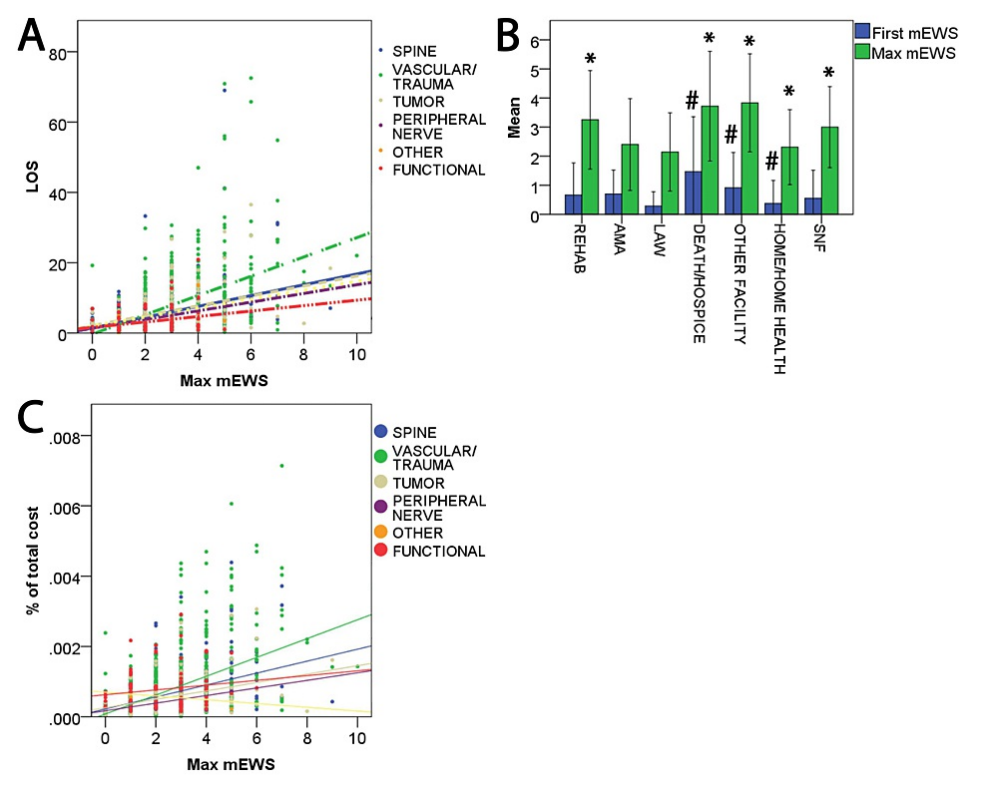
mEWS prediction of LOS, discharge disposition, and cost in neurosurgery (A) LOS linearly correlated with the maximum mEWS (greater correlation for vascular/trauma cases than other types of cases). (B) Increased likelihood of death was seen with a higher early mEWS (# indicates p < 0.05 comparing an early mEWS). A significant decrease in home/home-health disposition was seen with a higher maximum mEWS (* indicates p < 0.05 comparing a maximum mEWS). (C) Higher cost correlated with a higher maximum mEWS (greater correlation for vascular/trauma than other categories). mEWS, modified early warning score; LOS, length of stay; AMA, against medical advice; SNF, skilled nursing facility.

Patient outcomes were evaluated using the mEWS in univariate and multivariate analyses (Tables 4, 5 and Figure 2). On multivariate analysis, LOS was positively associated with CCI (OR = 1.05, 95% CI = 1.0-1.1, p = 0.05) and the maximum mEWS (OR = 1.8, 95% CI = 1.6-1.96, p = 0.0001). There was also a positive association seen for LOS and treatment categories, namely, among spine (OR = 2.7, 95% CI = 1.6-4.7, p = 0.0001), vascular/trauma (OR = 2.9, 95% CI = 1.7-4.8, p = 0.0001), tumor (OR = 3.2, 95% CI = 1.8-5.7, p = 0.0001), and peripheral nerve (OR = 3.1, 95% CI = 1.01-9.2, p = 0.05) groups (Tables 4, 5 and Figure 2A).

**Table 4:** Univariate analysis of outcomes using the modified early warning score (mEWS) OR, odds ratio; CI, confidence interval; CCI, Charlson Comorbidity Index; LOS, length of stay.

Variable	Length of stay		Routine disposition		Mortality		Higher patient cost	
	P-value	OR (95% CI)	P-value	OR (95% CI)	P-value	OR (95% CI)	P-value	OR (95% CI)
Age	0.4	1.0003 (0.997, 1.009)	0.0001	0.976 (0.969, 0.983)	0.02	1.03 (1.005, 1.05)	0.96	1.0 (0.99, 1.006)
CCI	0.001	1.07 (1.03, 1.1)	0.0001	0.86 (0.82, 0.89)	0.001	1.2 (1.1, 1.3)	0.5	1.01 (0.98, 1.05)
Sex, male	0.7	0.96 (0.78, 1.19)	0.4	0.9 (0.7, 1.1)	0.8	1.1 (0.5, 2.2)	0.6	1.07 (0.9, 1.3)
Category								
Spine	0.0001	2.8 (1.7, 4.7)	0.0001	0.3 (0.2, 0.6)	0.997	-	0.2	0.8 (0.5, 1.2)
Vascular/trauma	0.0001	3.3 (2.0, 5.4)	0.0001	0.3 (0.2, 0.6)	0.997	-	0.09	0.7 (0.4, 1.1)
Tumor	0.0001	3.5 (2.0, 5.9)	0.0001	0.2 (0.1, 0.4)	0.997	-	0.03	0.6 (0.4, 0.95)
Peripheral nerve	0.02	3.3 (1.2, 9.0)	0.07	0.3 (0.1, 1.1)	-	-	0.01	0.2 (0.08, 0.7)
Functional	Reference		Reference		Reference		Reference	
Other	0.3	2.96 (0.4, 22.2)	-	-	-	-	0.5	2.1 (0.2, 20.8)
LOS	-	-	0.0001	0.86 (0.83, 0.88)	0.2	0.9 (0.87, 1.03)	0.0001	1.5 (1.4, 1.6)
First mEWS	0.0001	1.3 (1.1, 1.4)	0.0001	0.7 (0.6, 0.8)	0.0001	1.9 (1.5, 2.3)	0.006	1.2 (1.05, 1.32)
Maximum mEWS	0.0001	1.8 (1.6, 1.9)	0.0001	0.7 (0.6, 0.7)	0.0001	1.5 (1.3, 1.8)	0.0001	1.6 (1.5, 1.8)

**Table 5:** Multivariate analysis of outcomes using the modified early warning score (mEWS) OR, odds ratio; CI, confidence interval; CCI, Charlson Comorbidity Index; LOS, length of stay.

Variable	Length of stay		Routine disposition		Mortality		Higher patient cost	
	P-value	OR (95% CI)	P-value	OR (95% CI)	P-value	OR (95% CI)	P-value	OR (95% CI)
Age	0.4	1.003 (0.996, 1.01)	0.0001	0.97 (0.961, 0.978)	0.06	1.02 (0.99, 1.05)	-	-
CCI	0.05	1.05 (1.0, 1.1)	0.0001	0.896 (0.854, 0.94)	0.007	1.16 (1.04, 1.3)	-	-
Sex, male	0.8	0.97 (0.77, 1.21)	-	-	0.6	1.2 (0.6, 2.6)	-	-
Category								
Spine	0.0001	2.7 (1.6, 4.7)	0.1	0.6 (0.3, 1.2)	-	-	0.0001	0.4 (0.2, 0.6)
Vascular/trauma	0.0001	2.9 (1.7, 4.8)	0.5	0.8 (0.3, 1.6)	-	-	0.0001	0.2 (0.1, 0.4)
Tumor	0.0001	3.2 (1.8, 5.7)	0.03	0.4 (0.2, 0.9)	-	-	0.0001	0.2 (0.1, 0.4)
Peripheral nerve	0.05	3.1 (1.01, 9.2)	0.4	0.5 (0.1, 2.2)	-	-	0.0001	0.07 (0.02, 0.3)
Functional	Reference		Reference				Reference	
Other	0.4	2.5 (0.3, 23.8)	-	-	-	-	0.8	1.5 (0.1, 21.4)
LOS	-	-	0.0001	0.87 (0.84, 0.9)	0.002	0.8 (0.8, 0.9)	0.0001	1.5 (1.42, 1.6)
First mEWS	0.5	0.95 (0.83, 1.1)	0.01	0.82 (0.71, 0.95)	0.001	1.5 (1.2, 2.0)	0.9	0.9 (0.8, 1.1)
Maximum mEWS	0.0001	1.8 (1.6, 1.96)	0.0001	0.8 (0.7, 0.9)	0.0001	1.7 (1.3, 2.1)	0.001	1.2 (1.1, 1.3)

After adjusting for other relevant variables, the factors associated with routine disposition were younger age (OR = 0.97, 95% CI = 0.961-0.978, p = 0.0001), lower CCI (OR = 0.896, 95% CI = 0.854-0.94, p = 0.0001), shorter LOS (OR = 0.87, 95% CI = 0.84-0.9, p = 0.0001), lower first mEWS (OR = 0.82, 95% CI = 0.71-0.95, p = 0.009), lower maximum mEWS (OR = 0.8, 95% CI = 0.7-0.9, p = 0.0001), and the tumor group (OR = 0.4, 95% CI = 0.2-0.9, p = 0.03) (Tables 4, 5 and Figure 2B). On multivariate analysis, higher mortality rates were associated with greater first mEWS (OR = 1.5, 95% CI = 1.2-2.0, p = 0.001), shorter LOS (OR = 0.8, 95% CI = 0.8-0.9, p = 0.002), greater CCI (OR = 1.16, 95% CI = 1.04-1.3, p = 0.007), and higher maximum mEWS (OR = 1.7, 95% CI = 1.3-2.1, p = 0.0001) (Tables 4, 5 and Figure 2B).

After adjusting for other covariates, cost was associated with longer LOS (OR = 1.5, 95% CI = 1.42-1.6, p = 0.0001) and higher maximum mEWS (OR = 1.2, 95% CI = 1.1-1.3, p = 0.001) (Tables 4, 5 and Figure 2C).

Machine learning algorithms

**Figure 3:**
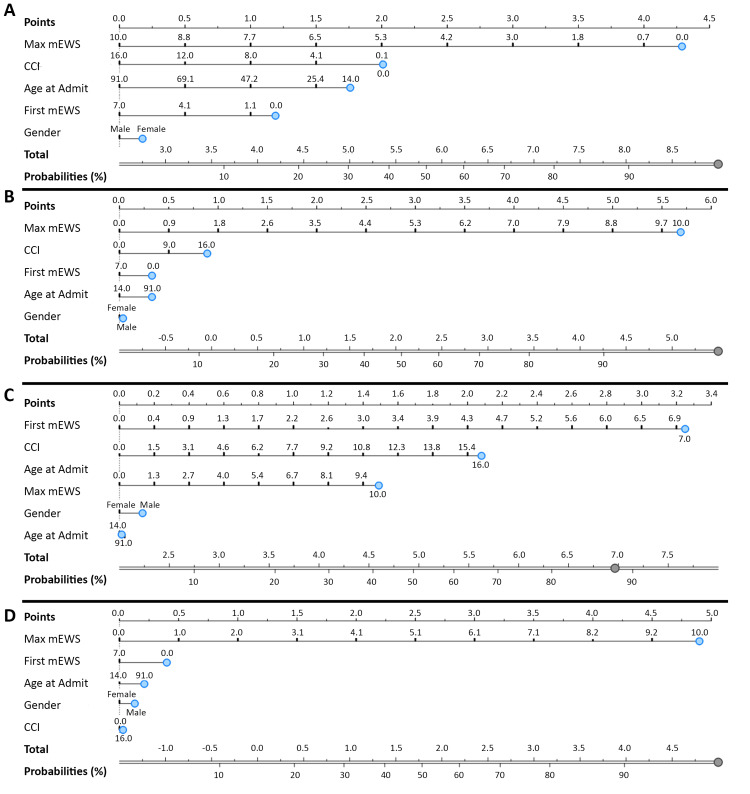
Nomograms using mEWS for predicting discharge disposition, LOS, mortality, and cost in neurosurgery patients Logistic regression nomograms were used to predict the probability of specific outcomes. A vertical line drawn upwards from the specific variable aligns with specific point values where the summation of total points indicated the probability of a specific outcome. (A) For discharge disposition, lower mEWS aligned with a higher probability of routine disposition. Increased (B) LOS, (C) mortality risk, and (D) cost were aligned with higher mEWS. The maximum mEWS was consistently the most pertinent variable except for the first mEWS in predicting mortality. mEWS, modified early warning score; LOS, length of stay; CCI, Charlson Comorbidity Index.

Predictive nomograms were generated using the mEWS for patient outcomes, allowing relative comparison of different factors, including routine disposition, longer LOS, increased mortality, and increased cost (Figure 3). New patients can have points sequentially added for each corresponding variable (e.g., maximum mEWS, CCI, age, first mEWS, and sex), and the sum of those points would correlate with a probability of outcome ranging from 0 to 100%. The maximum mEWS played the largest role in predicting outcomes as assessed by the greater contribution to the overall risk score and greater incremental space between specific values and scores.

**Figure 4:**
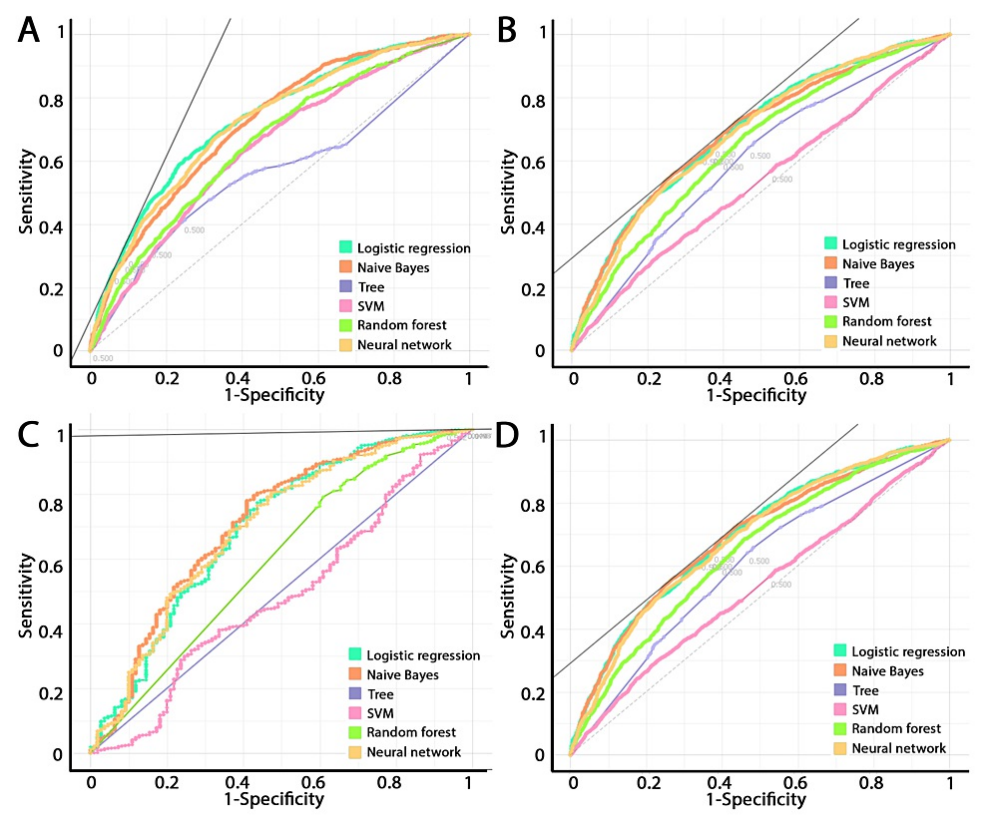
Receiver operating characteristic curves of various machine learning algorithms in predicting discharge disposition, LOS, mortality, and cost using the mEWS Logistic regression, naïve Bayes, and neural networks were the most predictive models for (A) discharge disposition (AUC range = 0.72-0.73), (B) LOS (AUC = 0.69-0.7), (C) mortality (AUC = 0.7-0.71), and (D) cost (AUC = 0.65-0.66). A tree algorithm with forward pruning, support vector machines, and random forest were less predictive. mEWS, modified early warning score; LOS, length of stay; AUC, area under the curve; SVM, support vector machine.

The machine learning algorithm prediction of outcome was compared by receiver operating characteristic (ROC) curves (Figure 4). Logistic regression, naïve Bayes, and neural networks were the most predictive across all outcomes. Tree algorithm with forward pruning, support vector machines, and random forest provided less robust predictive value.

## Discussion

Study findings

Although it may seem intuitive that elevated mEWS can predict worse outcomes in a neurosurgical population, this has not been quantified or evaluated before now. This study suggests that mEWS in the field of neurosurgery can be predictive of outcomes across a variety of neurosurgical procedures. Among all clinical factors, the maximum mEWS was the best predictor of outcome for discharge disposition, LOS, and total patient cost, and the first recorded mEWS was the best predictor of mortality. Moreover, the mEWS was instructive of outcomes across various diseases and after controlling for patient age and comorbidity. During the implementation of a mEWS-based quality improvement project at our institution, there were concerns among subspecialties that such a score did not apply to their specific patient populations; however, these results indicate that the mEWS can be used for this purpose in neurosurgery. The maximum mEWS was achieved on average within zero to one day of hospitalization, indicating its potential utility in improving patient outcomes if early interventions and monitoring are implemented as a result of the score. Unlike other studies showing a discrete cutoff for mEWS, our results suggested a continuous increase in morbidity with scores. Further study is needed to identify a cutoff to flag specific patient groups for interventions or closer care.

Predictive value of the mEWS

Over the past two decades, the use of the mEWS has been implemented by hundreds of hospitals [17,18]. Since its inception, the system has been used for a variety of purposes because of its efficacy as a predictive tool. It has been used to determine the need for a patient in the emergency department to be admitted to the hospital, to predict inpatient mortality rates, and to guide the postoperative care of surgical patients [12,17,19,20]. The use of the mEWS has also been associated with lower rates of intensive care unit (ICU) admissions, lower overall hospital costs, and a shorter LOS without an increase in mortality rates [13,21]. However, evidence to support the use of the mEWS in neurosurgery is new.

Multiple single-center studies have looked at different facets of the predictive value of the mEWS. Cei et al. [22] prospectively measured morbidity and mortality rates in 1,107 consecutive elderly patients using the mEWS and found that an elevated admission mEWS was significantly associated with higher rates of mortality and morbidity. Levin et al. [23] retrospectively evaluated 8,322 emergency department patients that had a combined ICU admission rate of 17% and an overall mortality rate of 2%. The authors reported that dynamic vital signs (evaluated with the mEWS) that were abnormal and failed to normalize were associated with increased rates of mortality, ICU admission, LOS, and sepsis diagnosis. The failure to normalize the mEWS was better associated with the outcome than any single score evaluated, namely, the triage mEWS, maximum mEWS, and last mEWS. Several studies have aimed to use the mEWS in this manner to identify high-risk patients and intervene early [13,24]. Our results support these findings suggesting that mEWS can predict poor outcomes in the general and subspecialty neurosurgery population.

One study also compared the CRP/albumin ratio to the mEWS and showed that the mEWS might be more sensitive in predicting mortality [25]. Our results support a correlation between inflammatory biomarkers and the mEWS but highlight the easier implementation of the mEWS into clinical decision-making. Kangas et al. [26] found that combining the systemic inflammatory response syndrome (SIRS) criteria with the mEWS resulted in correctly capturing three times more sepsis diagnoses while triggering 46% fewer false-positive alerts. Often biomarkers are used for evaluation of sepsis prior to culture results or in settings without clear symptoms. The use of mEWS may be a way to further improve the accuracy of sepsis in these neurosurgical patients.

Avenues for future development

This study suggests that further research into the combination of predictive tools, including those originally used for sepsis, is warranted to evaluate their effectiveness in different patient populations in an effort to improve safety and outcomes for patients and to further refine the predictive value of these tools. One finding of this study was the variability in utility for mEWS in specific neurosurgical patient populations, where a rise in mEWS distinctly impacted LOS and cost by patient type. Incorporation of the neurological examination into such a predictive score, for example, might be beneficial for neurosurgical patients because of the importance of the examination in surgical decision-making and its strong correlation with outcomes [27,28]. The addition of other biomarkers may also be a direction to improve predictive accuracy. The addition of imaging parameters and other neurosurgical patient classification could further refine patient prediction. Machine learning algorithms, briefly addressed in this study, may also be powerful in integrating large numbers of mEWS values across different time points. Such an algorithm could undergo real-time updates with any new data points during a patient’s hospitalization to predict any number of metrics.

Limitations of the mEWS

Limitations to the mEWS have been described in multiple studies. Cooksley et al. [29] performed a retrospective analysis of 840 patients at a specialist oncology hospital to assess the effectiveness of the mEWS to predict outcomes in oncology patients. ROC curves showed that the mEWS had a poor predictive value for admission to the ICU (0.55) and 30-day mortality (0.6). These data indicate that the mEWS may be limited in its ability to identify critical physiological parameters that contribute to outcomes in some cohorts, warranting ongoing evaluation and study among different patient populations. We felt our early results suggest mEWS could be helpful for a cohort of neurosurgical patients but needed further refinement. In addition, our results require further validation in other patient settings and institutions.

A limitation in our study involves the aggregation of multiple patient types into specific treatment categories, thus limiting understanding of specific patient procedures or diagnoses on the outcome. Further breakdown of patient procedures is required and planned for future studies. Implementing findings from the mEWS has been shown to improve survival after sepsis despite the variable presentation and sources of sepsis [13]. It is unclear whether and how prospectively implementing a mEWS protocol in neurosurgical patients would help improve outcomes in this population of patients beyond sepsis alone. This would be a question opportune for further study. As mentioned, incorporating neurological examinations and additional biomarkers in a neurosurgical mEWS may be a way of further improving the prediction of prognosis. Lastly, the specific components of the mEWS were integrated into a final score, and future work will aim to evaluate the impact of individual score components in relation to neurological examination findings or other biomarkers to assess for the most effective variables. Capturing these additional data in future studies could potentially help refine a neurosurgical mEWS.

## Conclusions

Our results suggest that the first recorded mEWS and the maximum recorded mEWS are highly predictive of disposition, LOS, mortality, and overall direct patient cost in a cohort of neurosurgical patients. The results suggested that real-time and dynamic physiologic alert systems could help distinguish patient risk early in a hospital course. These data add to the current literature involving the use of the mEWS as both an indicator of patient deterioration and a tool for predicting patient outcomes, and this study is the first evaluation of its use in a neurosurgical population. Understanding the ability of the mEWS to both improve outcomes as well as predict them across the entire spectrum of medical specialties is vital to proper patient care and management of hospitalized patients. Potential next steps would be further refinement of a predictive system, implementation of a real-time mEWS alert system among a neurosurgical population, and validation of a predictive algorithm in different patient settings or hospitals.
